# Biotechnological Approaches on Engineering Medicinal Tropane Alkaloid Production in Plants

**DOI:** 10.3389/fpls.2022.924413

**Published:** 2022-06-02

**Authors:** Haiyue Gong, Ping He, Xiaozhong Lan, Lingjiang Zeng, Zhihua Liao

**Affiliations:** ^1^School of Life Sciences, Integrative Science Center of Germplasm Creation in Western China (CHONGQING) Science City and Southwest University, The Provincial and Ministerial Co-founded Collaborative Innovation Center for R&D in Tibet Characteristic Agricultural and Animal Husbandry Resources, Southwest University, Chongqing, China; ^2^Chongqing Academy of Science and Technology, Chongqing, China; ^3^Xizang Agricultural and Husbandry College, The Provincial and Ministerial Co-founded Collaborative Innovation Center for R&D in Tibet Characteristic Agricultural and Animal Husbandry Resources, The Center for Xizang Chinese (Tibetan) Medicine Resource, TAAHC-SWU Medicinal Plant Joint R&D Centre, Nyingchi, China

**Keywords:** biosynthesis, biotechnology, metabolic engineering, synthetic biology, tropane alkaloids

## Abstract

Hyoscyamine and scopolamine, belonging to medicinal tropane alkaloids (MTAs), are potent anticholinergic drugs. Their industrial production relies on medicinal plants, but the levels of the two alkaloids are very low *in planta*. Engineering the MTA’s production is an everlasting hot topic for pharmaceutical industry. With understanding the MTA’s biosynthesis, biotechnological approaches are established to produce hyoscyamine and scopolamine in an efficient manner. Great advances have been obtained in engineering MTA’s production *in planta*. In this review, we summarize the advances on the biosynthesis of MTAs and engineering the MTA’s production in hairy root cultures, as well in plants. The problems and perspectives on engineering the MTA’s production are also discussed.

## Introduction

Medicinal plants constitute a treasure for human beings to obtain diverse drugs, flavors and fine chemicals. Of them, medicinal plants of Solanaceae produce a kind of specialized metabolites named tropane alkaloids (TAs), some of which have potent anticholinergic activity. Hyoscyamine and scopolamine are well-known medicinal tropane alkaloids (MTAs) and anticholinergic drugs (Grynkiewicz and Gadzikowska, 2008). They are clinically used to treat asthma, pain, motion sickness, functional gastrointestinal disorders, Parkinson’s syndrome and etc. (Huang et al., 2021). Furthermore, hyoscyamine is the key material for industrially producing ipratropium bromide, and scopolamine for tiotropium bromide. Ipratropium bromide, as well as tiotropium bromide, is an essential drug in the treatment of chronic obstructive pulmonary disease (COPD; Griffin et al., 2008). Therefore, the demand for the two alkaloids is huge. However, their production is limited due to low levels in medicinal plants producing TAs. To develop efficient ways to manufacture hyoscyamine and scopolamine, the scientists have been trying their best. Chemists successfully synthesized them, but failed in market because chemical synthesis was not economically availed (Grynkiewicz and Gadzikowska, 2008). Then, the final solution relies on biotechnological approaches, such as metabolic engineering and synthetic biology.

**Figure 1:**
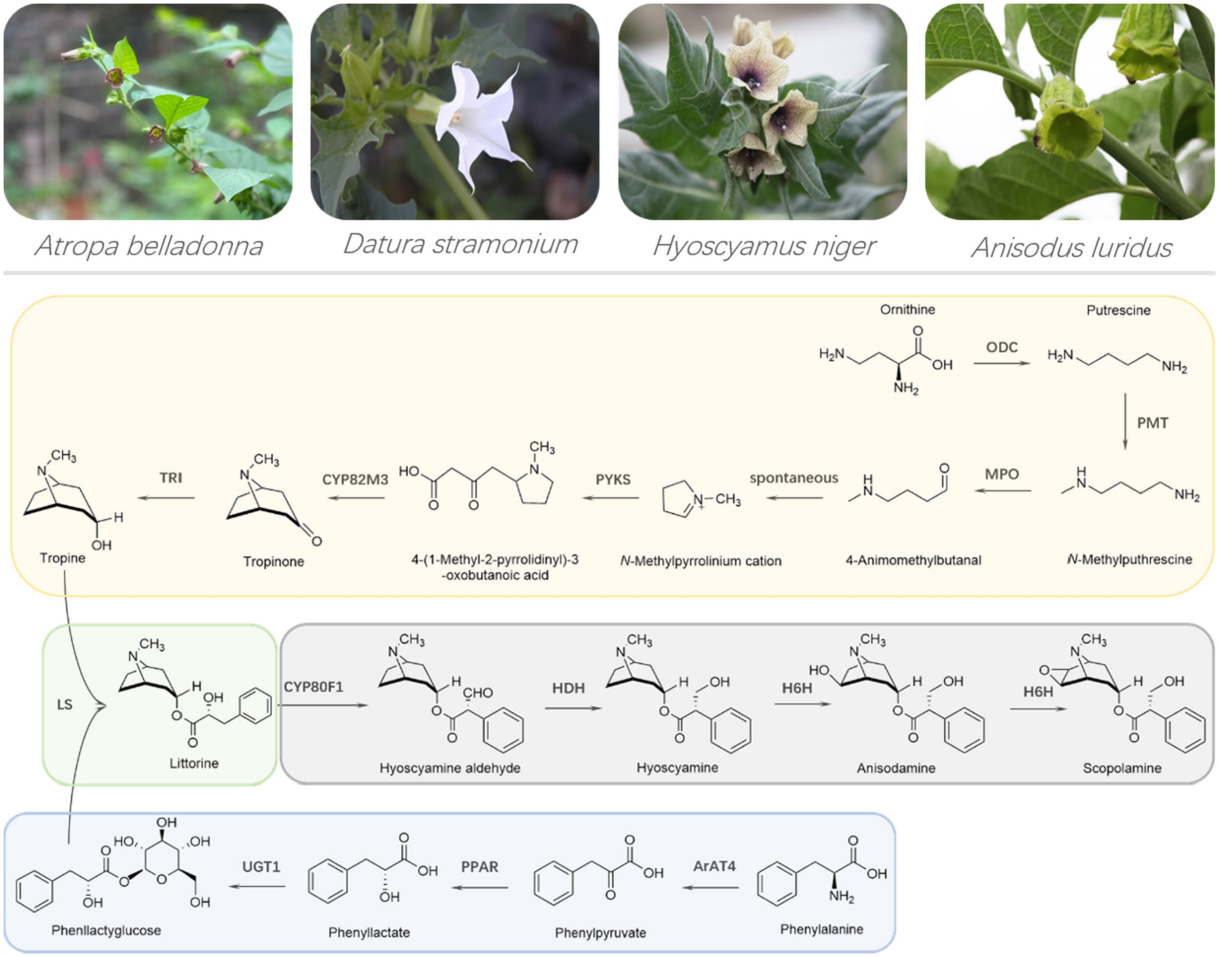
Representative plant species producing medicinal tropane alkaloids and the intact biosynthetic pathway of medicinal tropane alkaloids. ODC, ornithine decarboxylase; PMT, putrescine *N*-methyltransferase; MPO, *N*-methylputrescine oxidase; PYKS, polyketide type III synthase; CYP82M3, tropinone synthase; TRI, tropinone reductase I or tropine-forming reductase; ArAT4, phenylalanine aminotransferase; PPAR, phenylpyruvic acid reductase; UGT1, phenyllactate UDP-glycosyltransferase; LS, littorine synthase; CYP80F1, littorine mutase; HDH, hyoscyamine dehydrogenase or hyoscyamine aldehyde reductase; H6H, hyoscyamine 6*β*-hydroxylase.

Two amino acids, including ornithine and phenylalanine, are the starting precursors for the two MTAs, hyoscyamine, and scopolamine. The intact biosynthetic pathway of MTAs has been elucidated, which is composed of 13 biosynthesis enzymes (Figure 1). Such MTA’s biosynthesis enzymes and their corresponding genes provide valuable tools to engineering MTA’s production in plants and yeast cells. Although the production of hyoscyamine and scopolamine were obtained in engineered yeast cells, their liter yields are extremely low (Srinivasan and Smolke, 2020). For hyoscyamine and scopolamine, their highest production were, respectively, 80 and 30 μg L^−1^, very far away from the industry-requiring level, not less than 5 g L^−1^ (Srinivasan and Smolke, 2020). Therefore, the industrial production of MTAs is, and will be dependent on plant materials, not only at present but in a long future period. In this review, we summarize engineering MTA’s production in planta. The problems and perspectives on engineering MTA’s production are discussed, as well.

## Engineering Medicinal Tropane Alkaloid Production in Hairy Roots

Hairy root cultures are often used to engineering production of plant natural products. Hyoscyamine, as well as scopolamine, is specifically synthesized in secondary roots of plants (Li et al., 2006; Matthew et al., 2014; Qiu et al., 2021). A lot of publications report engineering the biosynthesis of MTAs in hairy root cultures of various MTA-producing medicinal plants. Overexpression of biosynthesis genes is a widely applied method to promote metabolite production. Seven biosynthesis genes required for MTA’s biosynthesis are studied in engineering the production of MTAs using hairy root cultures of different plants. Such biosynthesis genes include: *ornithine decarboxylase* (*ODC*), *putrescine N-methyltransferase* (*PMT*), *tropinone reductase I* (*TRI*), *littorine mutase* (*CYP80F1*), *hyoscyamine dehydrogenase* (*HDH*), and *hyoscyamine β-hydroxylase* (*H6H*).

### Engineering MTA’s Production by Overexpressing a Single MTA Biosynthesis Gene

Ornithine decarboxylase is the rate-limiting enzyme catalyzing the conversion of ornithine to putrescine (Bacchi et al., 1980). In *Atropa belladonna*, the ODC enzyme (AbODC) rather than arginine decarboxylase (AbADC) contributes to the MTA’s biosynthesis, and when *A. belladonna* ODC gene was overexpressed in hairy root cultures and plants of *A. belladonna*, the production of hyoscyamine and anisodamine was significantly increased (Zhao et al., 2020). Although ODC is located at the beginning of the MTA’s biosynthetic pathway, its role in promoting MTA’s production indicates that it is a key regulator directing ornithine into alkaloid metabolism (Zhao et al., 2020). Overexpression of *ODC* increases the putrescine level and provides metabolic flux enough for facilitating the downstream biochemical reactions (Zhao et al., 2020). However, such increased metabolic flux is not enough to support that *ODC* overexpression promotes MTA’s production. Another explanation might be polyamine metabolism regulated by *ODC* (Nölke et al., 2008). Polyamines are plant growth regulators involved in diverse cell physiology. Since the *ODC* enzyme is a key primary-metabolism one regulating polyamine biosynthesis, this implies that it may have complicated synergic effects on MTA’s biosynthesis through regulating polyamine biosynthesis.

Putrescine *N*-methyltransferase is a committed enzyme in catalyzing putrescine to form *N*-methylputrescine, and this enzyme is a secondary-metabolism enzyme (Hashimoto et al., 1989) Especially, tobacco PMT gene (*NtPMT*) were overexpressed in hairy root cultures of several MTA-producing plants, and such plants include *Duboisia* × hybrid (Moyano et al., 2002), *Hyoscyamus muticus* (Moyano et al., 2003), *Datura metal* (Moyano et al., 2003), *A. belladonna* (Rothe et al., 2003), *Hyoscyamus niger* (Zhang et al., 2004) and *Scopolia parviflora* (Lee et al., 2005). Overexpression of *NtPMT* in root cultures of *D*. hybrid, *A. belladonna*, and *H. niger* did not alter the production of hyoscyamine and scopolamine, while overexpression the same *PMT* gene (*NtPMT*) in hairy root cultures of *S. parviflora*, the levels of both hyoscyamine and scopolamine were significantly elevated. Moyano et al analyzed hyoscyamine and scopolamine content in individual clones of *H. muticus* and *D*. *metal* hairy root cultures with *NtPMT* overexpression, and they concluded that *NtPMT* overexpression increased hyoscyamine and scopolamine contents in *D*. *metal*, but only increased hyoscyamine content in *H. muticus*. Subsequently, Rothe et al reported their work on overexpression of *NtPMT* in hairy root cultures of *A. belladonna* and found no significantly increased production of MTAs when *NtPMT* was overexpressed. In the publication of Rothe et al. (2003), they discussed that the individual clones indicated both increased and decreased hyoscyamine and scopolamine content when *NtPMT* was overexpressed in root cultures of *H. muticus* and *Datura metel*. Zhang et al also found that overexpression of *NtPMT* did not promote hyoscyamine and scopolamine content in hairy root cultures of *H. niger* (Zhang et al., 2004). It should be noted that only two *NtPMT*-overexpressing root lines of *A. belladonna* and *S. parviflora* were used for studying MTA’s production by Rothe et al. (2003) and Lee et al. (2005). Probably, such a few root lines might not be enough to solidify their conclusion. Such different results above suggest the role of PMT in engineering MTA’s production should be carefully studied through intensive experiments.

Tropinone reductase I, also named tropinone-forming reductase, catalyzes tropinone reduction to give tropinone entering formation of littorine, a key intermediate for hyoscyamine biosynthesis (Hashimoto et al., 1992; Dräger, 2006). Richter et al. (2005) reported that overexpression of *Datura stramonium TRI* gene in hairy root cultures of *A. belladonna* markedly increased the TRI activity, tropine production, and contents of hyoscyamine and scopolamine. Kai et al found that overexpression *Anisodus acutangulus TRI* significantly increase the contents of hyoscyamine and scopolamine in hairy root cultures of *Ambrysus acutangulus*. Zhao et al also found that overexpression of *Scopolia lurida* (also named *Anisodus luridus*) *TRI* gene significantly promoted the production of hyoscyamine and scopolamine in hairy root cultures of *S. luridus* (Zhao et al., 2017). These previous reports indicate that TRI is a useful target on engineering MTA’s production in hairy root cultures, but there are not reports on *TRI* overexpression in transgenic plants.

Littorine mutase (CYP80F1) catalyzes littorine to form hyoscyamine aldehyde (Li et al., 2006), which is able to be reduced to give hyoscyamine under catalysis by hyoscyamine dehydrogenase (Qiu et al., 2021). The level of hyoscyamine aldehyde is extremely low in plants, suggesting that CYP80F1 might be a rate-limiting enzyme or hyoscyamine aldehyde might be metabolized very quickly to form some other related alkaloids. However, overexpression of *CYP80F1* showed no significant alteration in hyoscyamine and scopolamine production in hairy root cultures of *H. niger* (Li et al., 2006). Our group identified hyoscyamine dehydrogenase (Qiu et al., 2021), also named hyoscyamine aldehyde reductase (Liao et al., 2019), and its role in engineering MTA’s production was studied. When *A. belladonna* *HDH* gene was overexpressed in *A. belladonna* hairy root cultures, the production of hyoscyamine and scopolamine were markedly elevated, indicating that *HDH* is a valuable MTA biosynthesis gene in engineering MTA’s production (Qiu et al., 2021).

Hyoscyamine 6*β*-hydroxylase (H6H) catalyzes hyoscyamine to produce anisodamine through 6*β*-hydroxylation, and subsequently oxidizes anisodamine to generate scopolamine (Hashimoto and Yamada, 1986) The *H6H* genes have been identified from *H. niger* (Hashimoto and Yamada, 1986), *A. belladonna* (Li et al., 2012), *A. acutangulus* (Kai et al., 2011a), and *S. luridus* (Lan et al., 2018). Of these H6H enzymes, the *H. niger* one (HnH6H) showed the highest efficiency in converting hyoscyamine to scopolamine, and HnH6H is the best choice in engineering scopolamine production. Lan et al compared the effects of HnH6H and *S. luridus H6H* gene (*SlH6H*) on engineering scopolamine production, and found that HnH6H was much stronger than SlH6H in promoting scopolamine production (Lan et al., 2018). Without exception, overexpression of *H6H* definitively promoted the scopolamine content in hairy root cultures of different plants producing MTAs. However, the scopolamine production is quite a lot variable in engineered hairy root cultures generated from different plants, even when the same *H6H* gene (*HnH6H*) was overexpressed, indicating that the original capacity on MTA’s production depends on plant species themselves. Impressively, When *HnH6H* was overexpressed hairy root cultures of *H. muticus*, the best clone produce scopolamine at a level over 100 times more than the control clones (Jouhikainen et al., 1999). Of these *HnH6H*-overexpressing hairy root cultures, the highest scopolamine production was reported in *H. niger*, reaching 184 mg per liter (Zhang et al., 2004). However, it should be noted that hyoscyamine is not able to be completely converted to scopolamine in root cultures, even under *HnH6H* overexpression. To be concluded, overexpression of *H6H*, especially *HnH6H*, is absolutely necessary for engineering scopolamine production.

### Engineering MTA’s Production by Overexpressing Combinations of MTA Biosynthesis Genes

From ornithine and phenylalanine to hyoscyamine or scopolamine, more than 10 biosynthesis genes involved. Such a long biosynthetic pathway implies that there are several rate-limiting steps governing by some enzymes. Furthermore, individual MTA’s biosynthesis genes may have synergic effects on regulating alkaloid metabolism. Therefore, overexpression of the combinations of MTA’s biosynthesis genes is very important in engineering MTA’s production. Zhang et al. (2004) reported for the first time that overexpression of both *NtPMT* and *HnH6H* led to much higher levels of scopolamine in hairy root cultures than those overexpressing *NtPMT* or *HnH6H*. Kai et al. (2011b) also reported that the highest production of MTAs was obtained in *A. acutangulus* hairy root cultures overexpressing *AaPMT* and *AaTRI* together, compared with the scopolamine levels in those overexpressing *AaPMT* or *AaTRI*. Overexpression of both *AaTRI* and *AaH6H* also gave higher production of MTAs in hairy root cultures of *A. acutangulus*, than overexpression of *AaTRI* or *AaH6H* (Kai et al., 2012). Such overexpression of two MTA’s biosynthesis genes is able to be named a “Pushing-Pulling” strategy of metabolic engineering. When an upstream MTA’s biosynthesis gene, such as *PMT* before *TRI* and *H6H*, is overexpressed, its product as precursors for following enzymes is given more, and this pushes more metabolic flux to the downstream reactions. Simultaneously, when a downstream MTA’s biosynthesis gene is overexpressed, more precursors are able to be converted, showing a pulling force in biosynthetic pathway. To be concluded, overexpression of combination of MTA’s biosynthesis genes usually have better effects on promoting MTA’s production than that of a single gene.

Up to date, there are no reports on engineering MTA’s production through overexpression of three or more biosynthesis genes. Very recently, our group finished evaluation on *PYKS, CYP82M3, UGT1, LS*, two gene combinations (*PYKS + CYP82M3, UGT1 + LS*), and four gene combination (*PYKS + CYP82M3 + UGT1 + LS*), in engineering MTA’s production, and found interesting and valuable discoveries (submitted). According to the previously reported researches and our submitted ones, it can be draw a conclusion on simultaneous overexpression of several biosynthesis genes is a prior strategy in engineering MTA’s production. Although fruitful and marked achievements on engineering MTA’s production in hairy root cultures (Table 1), industrial production has not been successfully realized using hairy root cultures, because their cultivation in a large or industrial scale is not available technologically at present. So, it is necessary to develop engineered plants with high-yield MTA’s production.

**Table 1:** Engineering medicinal tropane alkaloid production in plant hairy root cultures through gene overexpression.

TA biosynthesis genes	Host plants	Metabolites	Content change	References
*AbODC*	*Atropa belladonna*	Putrescine	↑	Zhao et al., 2020
*mouse ODC*	*Datura innoxia*	Scopolamine	↑	Singh et al., 2011
*NtPMT*	*Datura metel*	Hyoscyamine	↑	Moyano et al., 2003
		Scopolamine	↑	
	*Hyoscyamus muticus*	Hyoscyamine	↑	
		Scopolamine	−	
	*Scopolia parviflora*	Hyoscyamine	↑	Lee et al., 2005
		Scopolamine	↑	
	*A. belladonna*	*N*-methylputrescine	↑	Rothe et al., 2003
		Hyoscyamine	−	
		Scopolamine	−	
	*Hyoscyamus niger*	Hyoscyamine	−	Zhang et al., 2004
		Scopolamine	−	
*SlTRI*	*Scopolia lurida* or *Anisodus luridus*	Hyoscyamine	↑	Zhao et al., 2017
		Scopolamine	↑	
*DsTRI*	*A. belladonna*	Hyoscyamine	↑	Richter et al., 2005
		Scopolamine	↑	
*HnCYP80F1*	*H. niger*	Hyoscyamine	−	Li et al., 2006
		Scopolamine	−	
*AbHDH*	*A. belladonna*	Hyoscyamine	↑	Qiu et al., 2021
		Anisodamine	↑	
		Scopolamine	↑	
*DiH6H*	*D. innoxia*	Hyoscyamine	↓	Li et al., 2020
		Scopolamine	↑	
*SlH6H*	*Scopolia lurida* or *Anisodus luridus*	Anisodamine	↑	Lan et al., 2018
		Scopolamine	↑	
*HnH6H*		Anisodamine	↑	
		Scopolamine	↑	
*HnH6H*	*H. muticus*	Scopolamine	↑	Jouhikainen et al., 1999
	*Hyoscyamus niger*	Scopolamine	↑	Zhang et al., 2004
	*Duboisia × hybrid*	Scopolamine	↑	Li et al., 2020
		Anisodamine	↓	
	*Scopolia parviflora*	Hyoscyamine	↑	Kang et al., 2005
		Scopolamine	↑	
*AaPMT + AaTRI*	*Anisodus acutangulus*	Hyoscyamine	↑	Kai et al., 2012
		Anisodamine	↑	
		Scopolamine	↑	
*AaTRI + AaH6H*	*Anisodus acutangulus*	Hyoscyamine	↑	Kai et al., 2012
		Anisodamine	↑	
		Scopolamine	↑	
*SpPMT + SpH6H*	*Scopolia parviflora*	Hyoscyamine	↑	Kang et al., 2011
		Scopolamine	↑	
*NtPMT + HnH6H*	*Atropa belladonna*	Hyoscyamine	↑	Yang et al., 2011
		Scopolamine	↑	
	*Hyoscyamus niger*	Scopolamine	↑	Zhang et al., 2004
	*Anisodus acutangulus*	Hyoscyamine	↑	Yan et al., 2014
		Scopolamine	↑	

↑, *increased production*; ↓, *decreased production*; −, no significant changes.

## Engineering Medicinal Tropane Alkaloid Production in Plants

To date, industrial production of medicinal tropane alkaloids is completely dependent on plant cultivation. So, engineered plants with high yield of hyoscyamine and scopolamine are highly desirable and valuable. For development of such engineered plants, genetic transformation with high efficiency is the most important technology. In fact, generated plants are reported in only a very few plant species producing MTAs, including *A. belladonna* and *Datura innoxia*.

Most of engineered plants with high-yield MTAs are reported in *A. belladonna* with overexpressing *HnH6H* for enhancement of scopolamine production. In 1992, transgenic plants of *A. belladonna* with *HnH6H* overexpression were developed by Yun et al. (1992). They established T0 progeny of *HnH6H*-overexpressing plants of *A. belladonna*, and further generated T1 progeny for analyzing the MTA’s production in different organs. The *HnH6H*-overexpressing plants of *A. belladonna* were grown under controlled conditions in a growth chamber. Surprisingly, hyoscyamine was almost completely converted to scopolamine in leaves and stems of T1-progeny plants with *HnH6H* overexpression, whereas only part of hyoscyamine was converted to scopolamine in roots. Such difference indicates that higher efficiency converting hyoscyamine to scopolamine in upground organs than that in underground organs. The scopolamine production was greatly elevated in all tested T1-progeny plants, and the highest scopolamine production was over 1.2% dry weight in leaves of the T1-12 line (Yun et al., 1992). Considering that synergic effects between PMT and H6H, our group generated *A. belladonna* plants, in which both *NtPMT* and *HnH6H* were overexpressed, and the scopolamine production was markedly increased as expected (Wang et al., 2011). Then, we further produced T1 progeny of *A. belladonna* plants with overexpression of both *NtPMT* and *HnH6H*, and investigated MTA’s production under the field conditions in the medicinal plant garden of Tibet Agriculture and Animal Husbandry University (Nyingchi, Tibet, China) and Xinpu Town of Guiyang City (Guizhou Province, China). Similar to the results obtained by Yun et al in *HnH6H*-overexpssing plants, hyoscyamine was completely converted to scopolamine in aerial organs and high production of scopolamine was detected. In the plants harvested from the field in Xinpu Town of Guiyang City, the highest production of scopolamine was 0.513% DW in leaves, indicating over 12 times higher than in wild-type levels (Xia et al., 2016). Similar results were obtained when *A. belladonna* plants with overexpressing both *NtPMT* and *HnH6H* (T1 progeny) were grown in the field of Tibet Agriculture and Animal Husbandry University, but the highest scopolamine level in leaves nearly reached 1% DW (Quan et al., 2016).

Compared with *HnH6H*-overexpressing plants generated by Yun et al, the scopolamine production in our transgenic plants was not so high, and possible reasons leading to such difference might have different genotypes of *A. belladonna* used as initial plant materials for transgenic research, different growth conditions, and some others. Notably, an obvious difference between Yun and our studies is the scopolamine production in roots. The scopolamine levels in secondary roots of *HnH6H-*overexpressing plants were even much lower than those in wild-type secondary roots, according to Yun et al. (1992). Without exception, overexpression of both *NtPMT* and *HnH6H* in greatly increased the scopolamine content of secondary roots in contrast to wild-type plants (Xia et al., 2016). It was postulated that synergic effects overexpressing both *NtPMT* and *HnH6H* facilitated the conversion from hyoscyamine to scopolamine in the native organ (secondary roots) synthesizing scopolamine (Zhang et al., 2004; Xia et al., 2016).

The biosynthesis genes including *TRI* and *ODC* were also used to engineering MTA’s production in transgenic plants. Zhao et al established transgenic plants of *A. belladonna*, in which the *ODC* gene of *A. belladonna* (*AbODC*) was overexpressed, and analyzed putrescine, *N*-methylputrescine, hyoscyamine, anisodamine and scopolamine in roots and leaves (Zhao et al., 2020). The production of putrescine, *N*-methylputrescine, hyoscyamine and anisodamine were significantly increased in roots of *AbODC*-overexpressing plants. The levels of putrescine, hyoscyamine, and anisodamine were also elevated in leaves of *AbODC*-overexpressing plants. The scopolamine production was not significantly altered in roots and leaves, even when *AbODC* was overexpressed. Overexpression of *AbODC* increased the production of hyoscyamine anisodamine, but did not promote scopolamine production, indicating that the epoxidation reaction catalyzed by *A. belladonna* H6H is a key limitation in converting anisodamine to scopolamine (Hashimoto and Yamada, 1986; Kluza et al., 2020). A mouse ODC gene was ectopically expressed in transgenic plants of *D. innoxia*, the polyamine and scopolamine levels were promoted, but hyoscyamine was not analyzed in this research (Singh et al., 2011). Although the scopolamine production was increased in transgenic plants of *D. innoxia*, the highest level for scopolamine was extremely low, 0.25 μg g^−1^ DW (Singh et al., 2011), suggesting that *D. innoxia* might be not a good candidate for industrially producing scopolamine. Rothe et al developed *NtPMT-*overexpressing plants of *A. belladonna*, and found that *NtPMT* overexpression did not increased the production of scopolamine, hyoscyamine, tropine, pseudotropine, tropinone, and calystegines. It should also be noted that there were only two transgenic plants used for analyzing alkaloid production (Rothe et al., 2003), and this might not be enough to draw a solidified conclusion, because the levels of these metabolites are variable in individual plants.

Notably, all the above transgenic plants with engineering MTA’s biosynthetic pathway are generated using kanamycin as selection pressure, given by neomycin phosphotransferase II (NPTII) as marker gene. As it is well known, the *NPTII* gene is useless when transgenic plants are obtained and it brings a biosafety risk for commercial usage of engineering plants. When transgenic plants with high-yield MTAs are developed, it is very important to establish transgenic homozygous lines that are highly valuable for agricultural and industrial application. However, no transgenic homozygous plants producing MTAs were reported before 2021. Therefore, our group took a few years to successfully develop transgenic homozygous plants of *A. belladonna*, with overexpression of both a glyphosate-resistant gene and *HnH6H*, and without antibiotics-resistant gene (Zhang et al., 2021). After obtaining T0 progeny with one-copy insertion, genetic breeding was used to develop homozygous offsprings. The field trials indicated that homozygous transgenic plants of *A. belladonna* grew well, resisted the commercial recommended concentration of glyphosate, produced high-yield scopolamine (7.04 mg g^−1^ DW in leaf). These homozygous transgenic plants of *A. belladonna* showed much higher commercial value than wild-type ones, because of their glyphosate resistance and high-yield scopolamine.

The CRISPR/Cas9 system is a powerful gene-editing tool, which is also used to generate *A. belladonna* plants with high-yield hyoscyamine (Zeng et al., 2021). Hyoscyamine is converted to anisodamine, and anisodamine to scopolamine under catalysis of H6H (Hashimoto and Yamada, 1986). Extracts of *A. belladonna* contain the three above alkaloids with similar structures, and it is expensive to separate each from total alkaloid extracts. Our group generated *A. belladonna* plants with disruption of the *H6H* gene through CRISPR/Cas9 system, the *H6H*-disrupting plants produced hyoscyamine at much higher levels than wild-type ones, because hyoscyamine cannot be converted to anisodamine and scopolamine, and they produced neither anisodamine nor scopolamine. The *H6H*-disrupting *A. belladonna* plants are valuable due to high-yield hyoscyamine and convenient separation of hyoscyamine.

Metabolic engineering of MTA’s production usually employs their biosynthesis genes. Very recently, our group has a publication on reporting engineering tropane alkaloid production using a novel calmodulin gene in *A. belladonna* plants (Zhang et al., 2022). Increased calcium can promote the TA’s production in plants, and accordingly, we postulated that calcium/calmodulin signaling might regulate TA’s biosynthesis and then identified a calcium-binding calmodulin gene (*AbCaM1*) highly expressed in secondary roots of *A. belladonna*. Transgenic plants of *A. belladonna*, in which both *AbCaM1* and *G2-EPSPS* were overexpressed, had strong tolerance to glyphosate and high-yield tropane alkaloids, including hyoscyamine, anisodamine and scopolamine. To our knowledge, this publication is the first report on engineering TA’s production in plants using a regulator protein. Such transgenic homozygous plants of *A. belladonna* are valuable for industrial production of medicinal TAs, due to their high production of TAs and strong tolerance to herbicide that facilitate manufacturing TAs at low cost.

## The problems on Engineering MTA’s Production in Plants

Although the MTA’s biosynthetic pathway has been completely unveiled, using *A. belladonna* as a model plant producing MTAs. The same enzyme from different MTA-producing plants may have various catalytic efficiency. For example, the H6H enzyme of *H. niger* shows much higher catalytic efficiency in converting hyoscyamine to scopolamine than the reported others. So, it is necessary to screen MTA’s biosynthesis enzymes with high catalytic efficiency from different plants producing MTAs. According to previous reports on enzymatic assays, it is suggested to pay more attention to the MTA’s biosynthetic enzymes, such as CYP80F1, phenyllactate UDP-glycosyltransferase and littorine synthase, each of which is a key limitation in the MTA’s biosynthesis (Bedewitz et al., 2018; Qiu et al., 2020). Furthermore, over 100 tropane alkaloids are reported in MTA-producing plants, and they compete against the biosynthesis of hyoscyamine and scopolamine. It is largely unknown on these alkaloid biosynthesis, so it is also important to understand their formation, and then genetic operation might be performed in planta to disrupt their biosynthesis and enhance the production of hyoscyamine and scopolamine.

To date, the values of most MTA’s biosynthesis genes are unknown in engineering MTA’s production in plants. It is of importance to have systematic evaluation on the values of MTA’s biosynthesis genes in plant metabolic engineering. And further, their combinations should also be tested in engineering MTA’s production. It can be reasonably predicted that combinations of MTA’s biosynthesis genes will be the main methods used in engineering MTA’s production in the future. Tens of solanaceous plant species are able to produce MTAs, and such abundant resources provide diverse plant chassis for engineering MTA’s production through metabolic engineering. However, of these MTA-producing plants, only the plants of *A. belladonna* can be efficiently transformed. Lack of genetic transformation at high efficiency is a key technological limitation for modification of MTA’s biosynthetic pathway in plants. So, plant biotechnology, especially transgenic technology, should be strengthened.

## The Prospects in Engineering MTA’s Production in Plants

Complete elucidation of MTA’s biosynthetic pathway results in synthesis of hyoscyamine and scopolamine in engineered yeast cells using the approaches of synthetic biology. Professor Smolke built the MTA’s biosynthetic pathway in yeast cells, and successfully synthesized hyoscyamine and scopolamine in engineered yeast cells (Srinivasan and Smolke, 2020). It is a milestone in manufacturing these valuable alkaloids. However, the production of hyoscyamine and scopolamine is extremely low, of which production levels are very far away from the standards of industrial production. Without doubt, the production of MTAs in engineered yeast cells will be gradually promoted, and probably it might reach or even exceed 5 g per liter, the lowest level for industrially producing TAs based on yeast’s fermentation. By the way, synthetic biology’s artemisinin fails in market is a typical case, and the most important reason for this failure is plant-base production of artemisinin is much cheaper than synthetic biology’s.

Surely, the plant biotechnologists have been trying their best to develop novel plant varieties producing MTAs at higher and higher levels, especially through complicated genetic operations composed of overexpression and suppression/gene edition methods. The next-generation strategy on engineering MTA’s production consists of enhancement of MTA’s production by overexpressing MTA’s biosynthesis genes, and simultaneously disruption of competitive metabolite’s production by suppressing or knocking out corresponding biosynthesis genes, in MTA-producing plants. Besides the core target on promoting MTA’s production, horticultural traits, such as biomass especially leaf yield, resistance on stress and pathogens and herbicide resistance, are able to be genetically fortified. Engineered plants, with increased MTA’s production and strengthened horticultural traits, will be the main source for industrial production of hyoscyamine and scopolamine, and engineered yeast cell factory may be an additional source for MTA’s production.

## Author Contributions

HG, LZ, and ZL conceived the original idea for the review, and edited and reviewed the manuscript. HG and PH drew the figure. XL and LZ checked the chemical structures and took the pictures of plant species. All authors wrote the manuscript. All authors contributed to the article and approved the submitted version.

## Funding

This work was financially supported by the NSFC projects (U1902212 and 31770335), the Fourth National Survey of Traditional Chinese Medicine Resources, Chinese or Tibet Medicinal Resources Investigation in Tibet Autonomous Region (State Administration of Chinese Traditional Medicine 20191217–540124 and 20200501–542329) and the College Students’ Innovative Entrepreneurial Training Plan Program (202110635108).

## Conflict of Interest

The authors declare that the research was conducted in the absence of any commercial or financial relationships that could be construed as a potential conflict of interest.

## Publisher’s Note

All claims expressed in this article are solely those of the authors and do not necessarily represent those of their affiliated organizations, or those of the publisher, the editors and the reviewers. Any product that may be evaluated in this article, or claim that may be made by its manufacturer, is not guaranteed or endorsed by the publisher.
